# Long-standing overt ventriculomegaly in adulthood with primary presentation of psychiatric disturbance: A case report

**DOI:** 10.1097/MD.0000000000027794

**Published:** 2021-12-10

**Authors:** Gao-jian Su, Jie Gao, Chu-wei Wu, Jun-feng Zou, Dong-liang Zhu, Jun Liu, Jie-hua Zhang, Xian-jian Huang

**Affiliations:** Shenzhen Key Laboratory of Neurosurgery, Department of Neurosurgery, the First Affiliated Hospital of Shenzhen University, Shenzhen Second People's Hospital, Shenzhen, China.

**Keywords:** adult, hydrocephalus, irritability, long-standing overt ventriculomegaly, ventriculoperitoneal shunt

## Abstract

**Rationale:**

Hydrocephalus is a common disease in neurosurgery. The typical symptoms of hydrocephalus include urinary incontinence, gait instability, and cognitive decline. Irritability rarely occurs in patients with hydrocephalus. Irritability rarely occurs in patients with hydrocephalus, especially in long-standing overt ventriculomegaly of adulthood (LOVA).

**Patient concerns:**

A 30-year-old female was admitted to our hospital because of mental retardation and unstable gait for more than 15 years. She had undergone ventriculoperitoneal shunt 15 years prior due to ventriculomegaly and related symptoms. However, the shunt catheter was removed shortly after surgery because of blockage, with no further postoperative treatment.

**Diagnosis:**

The patient was diagnosed with long-standing overt ventriculomegaly according to her head circumference and clinical symptoms, including adult hydrocephalus development, overt triventriculomegaly and absence of a secondary cause for aqueductal stenosis in adulthood.

**Interventions:**

After considerable discussion, she underwent ventriculoperitoneal shunt placement and showed dramatic and sustained improvement.

**Outcomes:**

The patient has been followed at 3-month intervals for over 2 years since discharge, and both the patient and family have reported a significant change in their daily life. She was able to live independently and control her emotions. Slight epilepsy was noted approximately 5 months after surgery but recovered 2 months later.

**Lessons:**

It is difficult to decide whether to treat LOVA when the in patients whose symptoms are not significant. We believe that early diagnosis and positive treatment can help improve outcomes and would recommend ventriculoperitoneal (VP) shunting in patients with LOVA.

## 1 Introduction

Hydrocephalus is a common disease in neurosurgery involving the symptomatic accumulation of cerebrospinal fluid (CSF) inside the cerebral ventricles.^[1]^ To date, the pathogenesis of hydrocephalus is not clear; its typical symptoms include urinary incontinence, gait instability, and cognitive decline.^[1–4]^ Hydrocephalus presents differently in adults and children with hydrocephalus. In previously published literature, physicians have used several terms, including “arrested hydrocephalus,” “asymptomatic hydrocephalus,” “occult hydrocephalus,” “compensated hydrocephalus,” “long-standing overt ventriculomegaly of adulthood” (LOVA), and “late-onset idiopathic aqueductal stenosis” to refer to these conditions.^[3]^

The term “LOVA” was first mentioned by Oi et al in the mid-1990s. It is a type of chronic hydrocephalus and presents in adults after a slow and drawn-out process, which begins at infancy, before the cranial suture is closed. In adulthood, symptoms occur years or decades later.^[5–7]^

However, the pathophysiology of intracranial pressure (ICP) elevation over such a long time remains unclear. Balevi et al indicated that decreased intracranial compliance along with relatively high ICP, is the pathophysiological basis of LOVA.^[4]^ Some physicians frequently considered this type of hydrocephalus to be a stable condition, and they often advocated conservative treatment and watchful waiting. However, some physicians considered it essential to perform shunt surgery, because progressive ventricular enlargement is life-threatening even after lengthy periods of stability.^[3]^ The optimal management of LOVA remains controversial. Therefore, careful selection is very important. We present a unique case of LOVA responsive to shunting in a patient with psychiatric dysfunction.

## 2 Clinical presentation

**Figure 1:**
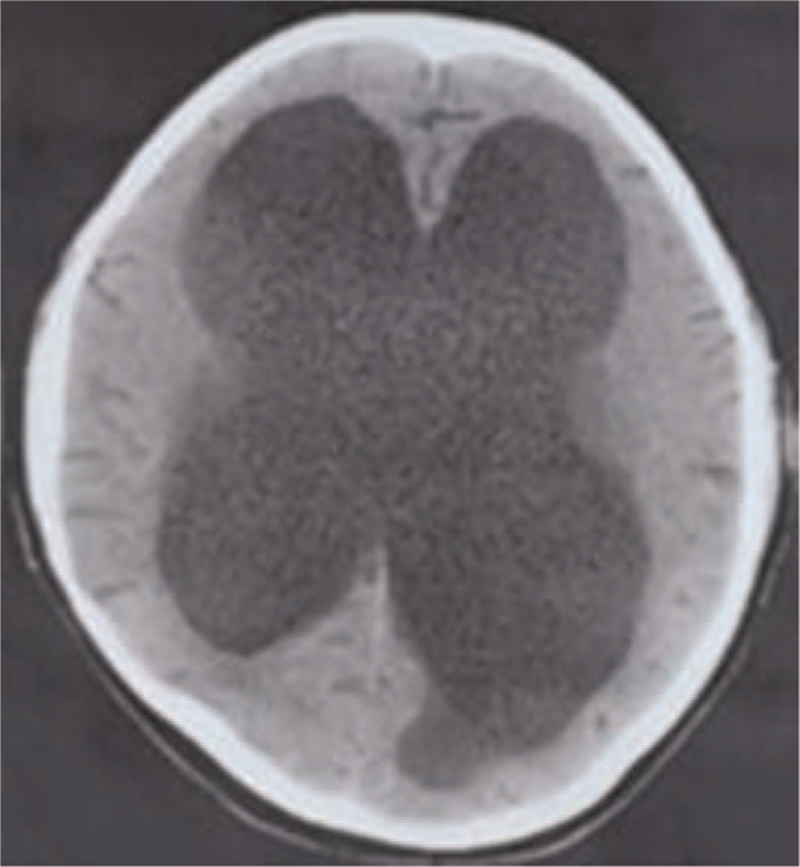
CT of the local hospital demonstrated that evident increase in ventricular size and reduction of sulci at parenchymal level.

A 30-year-old female presented to our hospital twice for treatment with a long history of progressive changes in function, especially in mental status. According to her family, her earliest manifestations 15 years earlier, were irritability, mental retardation, impaired verbal communication, and gait instability. However, they did not report but without urinary concerns. Computed tomography (CT) showed ventriculomegaly. The patient subsequently underwent a ventriculoperitoneal (VP) shunt in a local hospital, which failed due to a blockage soon after surgery. The shunt catheter was removed, but the patient did not receive further treatment until the symptoms worsened. She required considerable supervision and assistance in daily life = and was reported to be more irritable, but without any changes in urination. Furthermore, the CT performed at the local hospital, clearly showed severe ventriculomegaly (Fig. 1).

**Figure 2:**
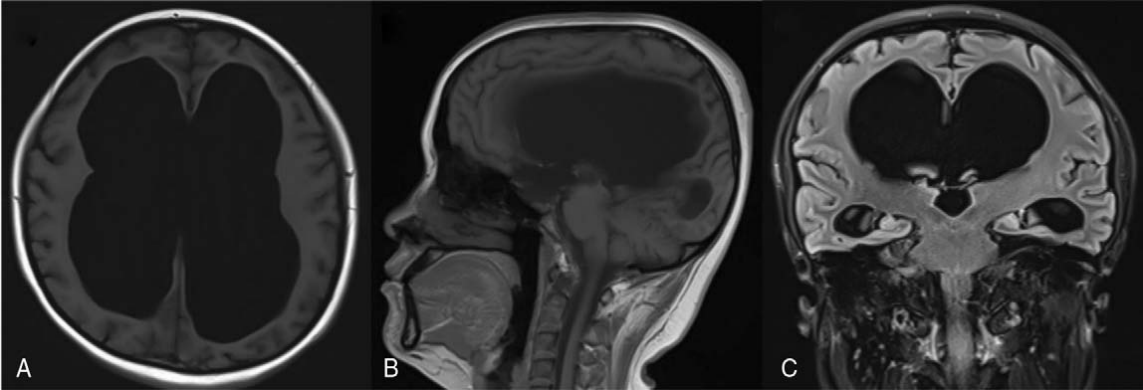
From left to right: T1-weighted magnetic resonance imaging in the transverse, coronal, and sagittal planes of the patient (A–C). Overt triventriculomegaly, cerebral aqueduct stenosis, and a concomitant severe ventriculomegaly are reported. Evans index: 0.66. Third ventricle width: 30 mm.

**Figure 3:**
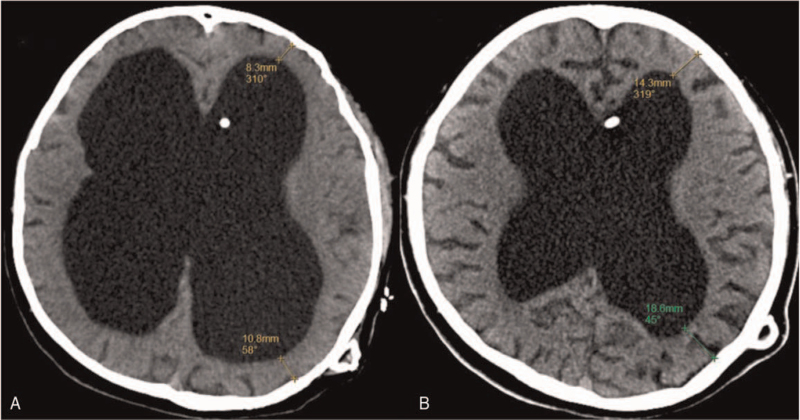
Preoperative and postoperative (2 years of follow-up) CT of the patient. CT demonstrated that the shortest distance from the anterior horn of the lateral ventricle to the skull was 8.3 mm and the shortest distance from the posterior horn of the lateral ventricle to the skull was 10.8 mm (A). CT demonstrated that the shortest distance from the anterior horn of the lateral ventricle to the skull was 14.3 mm and the shortest distance from the posterior horn of the lateral ventricle to the skull was 18.6 mm. The results showed that the ventricle size and ICP decreased, brain compliance increased and brain tissue improved. CT = computed tomography, ICP = intracranial pressure.

Physical examinations revealed that the patient was conscious and irritable but could cooperate with part of the examination. Her answers were occasionally inaccurate. The muscle strength levels of the right and left side extremities were 5 and 4, respectively. The head circumference was 53.0 cm. The Romberg sign was positive and Young's manic scale score was 18points. According to the preoperative evaluation, a lumbar puncture (LP) was performed with an observed opening pressure of 220 mmH_2_O. Initial nonenhanced magnetic resonance imaging showed overt triventriculomegaly, cerebral aqueduct stenosis, and severe ventriculomegaly (Fig. 2). After LP, the patient's symptoms improved. According to her head circumference and clinical symptoms, including adult hydrocephalus development, overt triventriculomegaly and absence of a secondary cause for aqueductal stenosis in adulthood, she was diagnosed with LOVA. Following exclusion of all contraindications, the patient underwent left VP shunt placement. Computed tomography obtained 1 month after surgery showed stable ventricle size with no subdural collections (Fig. 3A). Slight epilepsy was noted approximately 5 months after surgery but the patient recovered 2 months later. Beyond that, there were no events in the immediate postoperative period. Her shunt setting was lowered with good response, and she was still doing well at 2 years postoperatively. As time progressed, 2 years after surgery, CT revealed that the cortex became thicker (Fig. 3B).

The patient has been followed at 3-month intervals for over 2 years since discharge, and both the patient and family have reported a significant change in their daily lives. She was able to live independently and control her emotions. Physical examinations revealed that the patient was no apparent abnormalities and her Young's manic scale score was 2 points.

## 3 Discussion

Patients with hydrocephalus classically present with urinary incontinence, gait instability, and cognitive decline.^[1–4]^ Nevertheless, few studies have reported the primary presentation of psychiatric disturbances in patients with hydrocephalus, especially LOVA. Irritability was framed as having 2 components, tonic and phasic. Severe recurrent temper outbursts and irritable or angry mood between outbursts are the 2 components of irritability of disruptive mood dysregulation disorder.^[8]^ Irritability rarely occurs in patients with hydrocephalus. Some physicians consider that patients with hydrocephalus may suffer from brain damage with or without treatment. In addition, the prevalence, intensity, and persistence of irritability are still unknown or unconfirmed.^[9]^ Therefore, patients are administered conservative therapy. However, we believe that positive treatment, such as surgery, should be performed in patients with hydrocephalus. Overt ventriculomegaly was maintained in this patient for >15 years after shunt removal because of her family's lack of attention and knowledge. She underwent a left VP shunt, and the symptoms of irritability improved. Therefore, it seems that patients with LOVA may benefit significantly from positive treatment.

The normal surgical treatment of hydrocephalus involves VP, ventriculoatrial, and lumboperitoneal CSF shunts.^[10–13]^ The signs and symptoms of clinical deterioration due to an increase in CSF volume within the brain are indications for CSF shunting.^[14]^ Contraindications for CSF shunting include recent or impending abdominal surgery, recent peritonitis, and multiple abdominal adhesions.^[15]^ Endoscopic third ventriculostomy (ETV) has been widely used, and is a safe and reasonable alternative to patients with obstructive hydrocephalus.^[2,4,7,16–20]^ In the past, a history of meningitis was a contraindication for ETV. At present, it has been found that ETV can be used to treat hydrocephalus from etiologies like meningitis and tubercular meningitis.^[20]^ In addition, communicating hydrocephalus has traditionally been considered a contraindication for ETV, but its use has been investigated with promising results.^[20]^ Bleeding, injury of neural structures, hemodynamic changes, endocrine abnormalities and electrolyte imbalances, infection, and CSF leakage have been reported as complications of ETV surgery.^[21]^ Conversely, besides the ones listed for ETV, complications related to VPS include shunt obstruction, shunt malfunction, overdrainage and distal (abdominal) complications.^[20]^ Lu et al found that ETV can reduce the rate of complications because it avoids foreign body associated infections.^[22]^ On the basis of the meta-analysis of RCTs evaluating ETV and VP, ETV seems to be more beneficial for the patients of obstructive hydrocephalus, with lower rates of complications and mortality.^[22]^ It was found that VPS significantly reduced ICP and O_2_ saturation levels, and the ventricle size in hydrocephalus patients also decreased gradually.^[23]^ Furthermore, both ETV and VP experience failure; however, as time passes, the failure rate of VPS becomes lower than that of ETV.^[23]^

However, the optimal management for LOVA is unclear because of the lack of any randomized controlled trials.^[16]^ Investigators have debated the optimal CSF diversion procedure for decades.^[2]^ Some studies have advocated ETV for LOVA.^[2,4,7,16–19]^ However, further research is needed to evaluate the effectiveness of ETV in patients with LOVA. There is no evidence that ETV has an advantage over shunts.^[13]^ Fernando et al believe that a VP shunt is a superior method because it has better functional neurological outcomes 12 months after surgery than ETV.^[23]^ They considered that the complications of a VP shunt, including overdrainage and chronic subdural hematoma, could be avoided by using a programmable valve. Furthermore, patients who underwent ETV did not improve post-surgery and were predicted to undergo VP shunting. However, they could avoid secondary surgery by undergoing VP shunting initially.^[24]^ Kiefer et al reported a successful outcome in 87% of the cases with a 12% complication rate in a 26 patients cohort treated with gravitational valves in VP.^[5]^ In 1 series, 6 patients with LOVA underwent ETV. All of them required a secondary procedure. Ono et al^[25]^ deemed that adding one more programmable pressure valves was effective and appears to be a useful choice for treating overdrainage. In recent time, various ventriculoperitoneal shunts were designed to prevent overdrainage.^[26]^ Further studies are needed to determine which shunt is the most appropriate for LOVA. We believe that patients with LOVA can benefit more from VP shunt surgery. Our patient had a profound response to shunting with a longer follow-up period.

We present a unique case of LOVA responsive to shunting in a patient with psychiatric dysfunction. It is difficult to decide whether to treat LOVA in patients whose symptoms are not significant. We believe that early diagnosis and positive treatment can help improve outcomes and would recommend VP shunting in patients with LOVA.

## 4 Patient perspective

The patient was satisfied with our treatment and expressed her sincere appreciation for our help.

## Acknowledgments

The authors thank the physicians and nurses of the Shenzhen Second People's Hospital Department of Neurosurgery.

## Author contributions

JG, CWW, and JFZ collected the data. DLZ, JL, and JHZ interpreted the data. GJS and XJH wrote the first draft of the manuscript. All authors commented on the manuscript and have read and approved the final version.

**Conceptualization:** Gao-jian Su, Jie Gao, Chu-wei Wu, Jun-feng Zou, Dong-liang Zhu, Jun Liu, Jie-hua Zhang.

**Data curation:** Gao-jian Su, Jie Gao, Chu-wei Wu, Jun-feng Zou, Dong-liang Zhu, Jun Liu, Jie-hua Zhang.

**Investigation:** Gao-jian Su.

**Supervision:** Xian-jian Huang.

**Validation:** Xian-jian Huang.

**Writing – original draft:** Gao-jian Su, Xian-jian Huang.

**Writing – review & editing:** Xian-jian Huang.
